# Polymorphic Variants of Selected Genes Regulating Bile Acid Homeostasis in Women with Intrahepatic Cholestasis of Pregnancy

**DOI:** 10.3390/ijms26157456

**Published:** 2025-08-01

**Authors:** Krzysztof Piątek, Grażyna Kurzawińska, Marcin Ożarowski, Piotr Józef Olbromski, Adam Kamiński, Maciej Brązert, Tomasz M. Karpiński, Wiesław Markwitz, Agnieszka Seremak-Mrozikiewicz

**Affiliations:** 1 Department of Gynecology and Obstetrics, University of Zielona Gora, Licealna 9, 65-417 Zielona Gora, Poland; krzysztofpiatek.jr@gmail.com; 2 Laboratory of Molecular Biology, Department of Perinatology, Poznan University of Medical Sciences, Polna 33, 60-535 Poznan, Poland; gkurzawinska@ump.edu.pl (G.K.); asm@data.pl (A.S.-M.); 3 Department of Biotechnology, Institute of Natural Fibres and Medicinal Plants—National Research Institute, Wojska Polskiego 71B, 60-630 Poznan, Poland; 4 Clinic of Operational Gynecology, Poznan University of Medical Sciences, Polna 33, 60-535 Poznan, Poland; olbromski.piotr@gmail.com; 5 Department of Orthopedics and Traumatology, Independent Public Clinical Hospital No. 1, Pomeranian Medical University in Szczecin, Unii Lubelskiej 1, 71-252 Szczecin, Poland; adam.kaminski@pum.edu.pl; 6 Department of Diagnostics and Treatment of Infertility, Poznan University of Medical Sciences, Polna 33, 60-535 Poznan, Poland; maciejbrazert@gmail.com; 7 Chair and Department of Medical Microbiology, Poznan University of Medical Sciences, Rokietnicka 10, 60-806 Poznań, Poland; 8 Department of Perinatology, Poznan University of Medical Sciences, Polna 33, 60-535 Poznan, Poland; wmarkwitz@ump.edu.pl

**Keywords:** polymorphic variants, genes regulating bile acid homeostasis, intrahepatic cholestasis, pregnancy

## Abstract

Intrahepatic cholestasis of pregnancy (ICP) is characterized by the onset of pruritus and elevated serum transaminases and bile acids (BA). The key enzyme in BA synthesis is CYP7A1, and its functions are regulated by various nuclear receptors. The goal of this study is to evaluate the association between *CYP7A1*, *NR1H1*, *RXRA*, and *PPARA* gene variants and risk of ICP. Five single nucleotide variants (SNVs), rs3808607 (*CYP7A1*), rs56163822 (*NR1H4*), rs1800206 (*PPARA*), rs749759, and rs11381416 (*NR2B1*), were genotyped in a group of 96 ICP and 211 controls. The T allele of the *CYP7A1* (rs3808607) variant may be a protective factor against ICP risk (OR = 0.697, 95% CI: 0.495–0.981, *p* = 0.038). Genetic model analysis showed that rs3808607 was associated with decreased risk of ICP under dominant (OR = 0.55, 95% CI: 0.32–3.16, *p* = 0.032, AIC = 380.9) and log-additive models (OR = 0.71, 95% CI: 0.51–1.00, *p* = 0.046, AIC = 381.4). The A insertion in the rs11381416 *NR2B1* variant was associated with the degree of elevation in the liver function tests TBA (34.3 vs. 18.8 μmol/L, *p* = 0.002), ALT (397.0 vs. 213.0 IU/L, *p* = 0.017), and AST (186.0 vs. 114.4 IU/L, *p* = 0.032) in ICP women. Results indicate an association between the *CYP7A1* rs3808607 and the risk of ICP and the association of the rs11381416 of the *NR2B1* receptor with higher values of liver function tests in women with ICP. A better understanding of the cooperation of proteins involved in BA metabolism may have important therapeutic implications in ICP and other hepatobiliary diseases.

## 1. Introduction

ICP (Intrahepatic cholestasis of pregnancy) is a liver disorder that occurs in the late second and early third trimester of pregnancy. Although ICP is associated with relatively mild complications for the mother, such as intense itching and liver dysfunction, it may increase the risk of later hepatobiliary disease. However, the condition carries serious risks for the fetus, including preterm labor, meconium-stained amniotic fluid, and stillbirth [1,2]. The study by Floreani et al. [3] showed an increased risk of preterm birth (*p* < 0.001) and a higher rate of cesarean delivery (*p* = 0.05) [3]. A prospective cohort study from Sweden showed that the probability of fetal complications (spontaneous preterm deliveries, asphyxial events, and meconium staining of amniotic fluid, placenta, and membranes) increased by 1–2% per additional μmol/L of serum bile acids. However, the researchers emphasize that the increased risk to the fetus applies to patients with ICP whose bile acid concentration was <40 μmol/L [4]. In the study by Kawakita et al. [5] (2015), total bile acid (TBA) concentrations of 40–99.9 μmol/L and TBA ≥ 100 μmol/L were associated with an increased risk of meconium in the amniotic fluid (adjusted OR = 3.55 and OR = 4.55, respectively) [5].

The etiology of ICP is complex and not fully understood. The development of ICP is believed to be the result of many factors, such as genetic predisposition, hormonal factors, environmental factors and nutritional deficiencies, as well as the influence of chronic diseases on the predisposition to the development of cholestasis [6,7,8,9]. ICP causes abnormalities in laboratory test results of liver enzymes: aminotransferases: aspartate, alanine and γ-glutamyl transpeptidase (ASP, ALT and GGT) and increased values of bile acids (BAs). There are no universal consensus and guideline for the baseline of TBA for the diagnosis of ICP; however, the most common is serum level ≥ 10 μmol/L [10].

Bile acids are essential for the solubilization, digestion and absorption of dietary lipids and fat-soluble vitamins in the small intestine and are a signal molecule that regulates lipid, glucose and energy homeostasis. BAs regulate such many processes through activation of receptors including the farnesoid X receptor (FXR), the vitamin D receptor (VDR), the pregnane X receptor (PXR), membrane-bound G protein-coupled receptor Takeda G protein-coupled receptor 5 (TGR5), α5 β1 integrin, and sphingosine-1-phosphate receptor 2 (S1PR2). Several of these receptors are expressed outside of the gastrointestinal system, indicating that bile acids may have diverse functions throughout the body [11,12]. Bile acids are synthesized predominantly in the liver through the enzymatic oxidation of cholesterol via two different routes. The main pathway, which accounts for about 75% of BA production, is called the classical or neutral. This pathway is initiated by the rate-limiting enzyme, cholesterol 7alpha-monooxygenase (CYP7A1, EC:1.14.14.23), which catalyzes the hydroxylation of cholesterol to 7alpha-hydroxycholesterol. Cholesterol 7alpha-monooxygenase is encoded by the cytochrome P450 family 7 subfamily A member 1 gene (*CYP7A1* Gene ID:1581) located on chromosome 8, regions q11–12, spans 10 kb and contains 6 exons and 5 introns [13]. Single nucleotide variant (SNV) in the *CYP7A1* gene at the—204 location from the transcriptional start site (the—278 location from the translation initiation codon), conferring an A to C variation, may play a critical role in gene expression, leading to altered enzyme levels (rs3808607: the forward allele orientation G > T) [14,15]. This variant has been shown to influence serum levels of total cholesterol, LDL, and triglycerides [16,17].

The transcriptional regulation of the *CYP7A1* gene is complex and involves numerous nuclear receptors (NRs). Farnesoid X receptor (*FXRA*, *NR1H4*, Gene ID: 9971, further on referred to as “*FXR*”) was the first NR identified as a bile acid receptor and was shown to regulate the transcription of key genes in bile acid metabolism, from synthesis and transport to detoxification [18,19]. The expression of the gene encoding FXR has been demonstrated in the adrenal glands, adipose tissue, endothelial wall, pancreas and kidneys. However, it is particularly high in the liver and intestine, which indicates an important role of this receptor in the regulation of enterohepatic circulation of BA [20]. A common *FXR* genetic variant rs56163822 (−1G > T), a SNV adjacent to the ATG start codon located within the Kozak consensus motif of the *FXR* gene, is linked to reduced transactivation of *FXR* gene targets [21]. Since the Kozak consensus motif ensures ribosomal binding to mRNA transcripts and efficient protein translation, genetic variation in this conserved sequence is associated with decreased protein translation [22,23].

**Figure 1:**
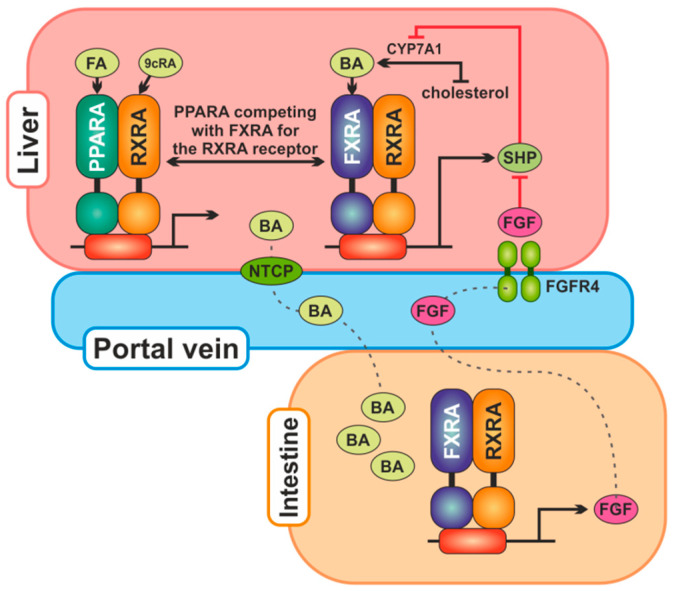
The main relationships between proteins encoded by genes analyzed in the work. Bile acids (BA) are produced in the liver from cholesterol by CYP7A1 and secreted into the gallbladder. From the ileum, they are reabsorbed in enterocytes, where they activate FXR, which stimulates transcription of the *FGF19* gene. The FGF19 protein is secreted into the portal circulation and in the liver binds itself to the receptor FGFR4 to reduce CYP7A1 expression. Bile acids synthesis in the liver is also regulated by FXR, which transcriptionally increases SHP protein expression, which reduces CYP7A1 expression. PPARA competes with FXR for the RXRA receptor and may downregulate SHP expression. Abbreviations: BA, bile acids; FA, fatty acids; CYP7A1, cholesterol 7alpha-monooxygenase; FGF, fibroblast growth factor; FGFR4, fibroblast growth factor receptor 4; FXRA, farnesoid X receptor alpha; NTCP, Na^+^-taurocholate cotransporting polypeptide; PPARA, peroxisome proliferator-activated receptor alpha; RXRA, retinoid X receptor alpha; SHP, small heterodimer partner; 9cRA, 9-cis retinoic acid.

FXR can regulate enterohepatic circulation by acting on BA transporters: apical sodium-dependent BA transporter (ASBT, SLC10A2), organic solute transporter-α and -β (OST-α, SLC51A; OST-β, SLC51B), Na^+^-taurocholate cotransporting polypeptide (NTCP, SLC10A1) and bile salt export pump (BSEP, ABCB11). FXR also regulates BA synthesis via two pathways, one in the liver by the small heterodimer partner 1 protein (SHP-1) and the other in the intestine involving fibroblast growth factor FGF15/19 (FGF15 in rodents; FGF19 in humans) [24]. As a transcription factor, FXR binds to DNA either as a monomer or as a heterodimer with a common partner for NRs, retinoid X receptor (*RXRA*, *NR2B1* Gene ID: 6256), to regulate the expression of various genes involved in bile acid (BA), lipid, and glucose metabolisms [25,26]. Peroxisome proliferator-activated receptors (PPARs), a group of nuclear receptor proteins, also play an active role in the regulation of bile acid metabolism. Three types of PPARs have been identified: alpha, gamma, and delta (beta). Activation of *PPARA* induces *CYP8B1* expression and inhibits *CYP7A1* expression in the classical pathway; whereas in the alternative pathway, it inhibits *CYP7B1* and *CYP27A1* expression. The alpha isoform can also inhibit the FXR signaling pathway by competing with FXR for the RXRA receptor, can affect the FXR/SHP-1 pathway by inhibiting SHP-1 expression, and can increase the expression of bile acid transport proteins such as BSEP, MRP3, and MRP2 [24,27,28]. *PPARA* (*NR1C1*, Gene ID: 5465) is located on 22q13.3, and several single nucleotide polymorphisms described within this gene were associated with metabolic features like insulin resistance, dyslipidemia and cardiovascular risk factors. The rs1800206 variant is found in the fifth exon, which is a result of a transversion (C > G) in exon 5 that alters the amino acid sequence of the PPARA protein at the 162 codon (Leucine to Valine, L162V) [29,30]. The main relationships between proteins encoded by genes analyzed in the work are presented in Figure 1.

The goal of this study is to evaluate the association between polymorphic variants of the *CYP7A1* gene, which is crucial for the synthesis of bile acids, and selected nuclear receptor genes (*FXR*, *RXRA* and *PPARA*) regulating its function in women with ICP.

## 2. Results

### 2.1. Characteristics of the Study Population

The present research included 96 ICP patients and 211 controls. General characteristics of study subjects are listed in Table 1, including age, body mass index (BMI), blood pressure, obstetric data of patients and selected clinical parameters of their newborns. Patients from both groups were of comparable age (30.43 ± 4.24 vs. 30.68 ± 4.67 in controls, *p* = 0.648), and there were no differences in blood pressure and BMI before pregnancy. More than half of women with ICP (56.26%) experienced preterm labor (<37 weeks) (*p* < 0.001). The median of pregnancy termination was two weeks lower in the ICP group compared to the controls (37 vs. 39 weeks, *p* < 0.001), which likely contributed to the observed smaller increase in BMI during pregnancy in this group (4.11 vs. 5.21 kg/m^2^, *p* < 0.001). Gestational weight gain (GWG) was calculated based on Institute of Medicine (IOM) guidelines [31] related to pre-pregnancy BMI: underweight, a gain of 12.5–18 kg; normal weight, a gain of 11.5–16 kg; overweight, a gain of 7–11.5 kg; and obese, a gain of 5–9 kg. In the cholestasis group, there were more women with insufficient body weight gain (41.67% vs. 24.64% in controls); normal and excessive body weight gain during pregnancy was more often observed in controls (*p* = 0.010). In women with cholestasis, pregnancies were more often terminated by surgical delivery (45.83% vs. 32.70% in controls, *p* = 0.037). We found a statistical difference between the case and the controls concerning the newborn weights (3091.03 ± 634.81 g vs. 3425.59 ± 433.82 g in controls, *p* < 0.001), whereas the mean for placental weight was lower in the ICP group (581.06 ± 150.63 g vs. 620.18 ± 111.37 g), but without statistical significance (*p* = 0.053). In patients with ICP, the median serum level of TBA was 19.75 [IQR: 14.93; 33.36] µmol/L. In most (79.17%), TBA values were below 40 µmol/L; in 17.71%, they were above; and in three women (3.12%), they reached values above 100 µmol/L. Pruritus was present in 93% of women, none of whom had jaundice.

**Figure 2:**
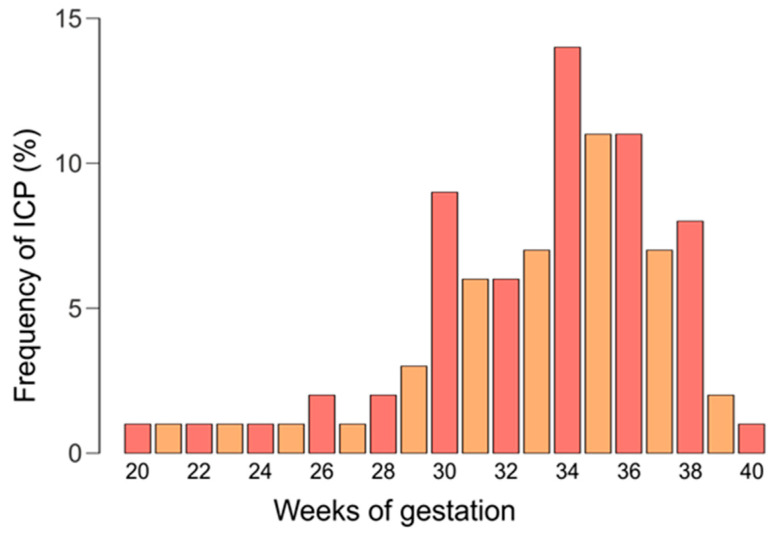
The gestational time of onset of intrahepatic cholestasis symptoms.

In 72% of women, ICP symptoms appeared between 28 and 36 weeks of pregnancy, in 9%, in the second trimester of pregnancy (<28 weeks), and in 19%, after 37 weeks. The gestational time of onset of intrahepatic cholestasis symptoms in the study group is presented in Figure 2.

### 2.2. Genetic Association Analyses

We found no deviation from Hardy–Weinberg equilibrium (HWE) in controls regarding all studied SNVs (*p* > 0.05). The MAF (minor allele frequency) of rs3808607 in cases was significantly lower than controls (47.3% vs. 56.3%), which suggested the T allele of this *CYP7A1* variant may be a protective factor against ICP risk (OR = 0.697, 95% CI: 0.495–0.981, *p* = 0.038) (Table 2).

The association between selected SNVs in ICP groups and controls was analyzed with logistic regression and presented in Table 3. Of the five genetic variants analyzed in this work, only rs3808607 *CYP7A1* showed a statistically significant association with ICP. The GG genotype was more frequent in women with ICP compared to the control group (31.3% vs. 19.9%). Genotypes containing at least one T allele reduce the risk of ICP in the studied female population (GT: OR = 0.57, 95% CI: 0.32–1.04 and TT: OR = 0.51, 95% CI: 0.26–0.98, *p* = 0.093, AIC = 382.7). Under the dominant (OR = 0.55, 95% CI: 0.32–3.16, *p* = 0.032, AIC = 380.9) and log-additive (OR = 0.71, 95% CI: 0.51–1.00, *p* = 0.046, AIC = 381.4) models, the rs3808607 was significantly associated with a lower risk of ICP occurrence.

### 2.3. Serum TBA Level Stratified Analysis

Patients with ICP were divided into two groups based on TBA levels below (N = 76) and above 40 μmol/L (N = 20) according to guidelines of the Royal College of Obstetricians and Gynaecologists (RCOG). These guidelines classify ICP as mild (19–39 μmol/L), moderate ICP (40–99 μmol/L), and severe ICP (>100 μmol/L) [32].

Comparing the clinical data of the patients and their children, no statistically significant differences were observed between the groups. The genotype and allele frequencies rs3808607 (*CYP7A1*), rs56163822 (*NR1H4*), rs1800206 (*PPARA*), and rs749759 (*NR2B1*) were also not statistically significantly different. For rs11381416 *RXRA* gene, we found that the genotypes with A insertion are more frequent in women with TBA ≥ 40 μmol/L compared to the second subgroup (dominant model: 30.0% vs. 11.8% in group with TBA below 40 μmol/L, OR = 3.19, 95% CI: 0.98–10.41, *p* = 0.062, AIC = 98.8).

### 2.4. The Gestational Time of Onset of ICP Symptoms Stratified Analysis

We divided the study group into three subgroups based on the time of onset of ICP symptoms: before the third trimester (<28 weeks), early third trimester (28 to 36 weeks), and late third trimester (≥37 weeks). When analyzing the frequency of genotypes and alleles of the studied SNVs in such subgroups, no statistically significant differences were observed.

**Figure 3:**
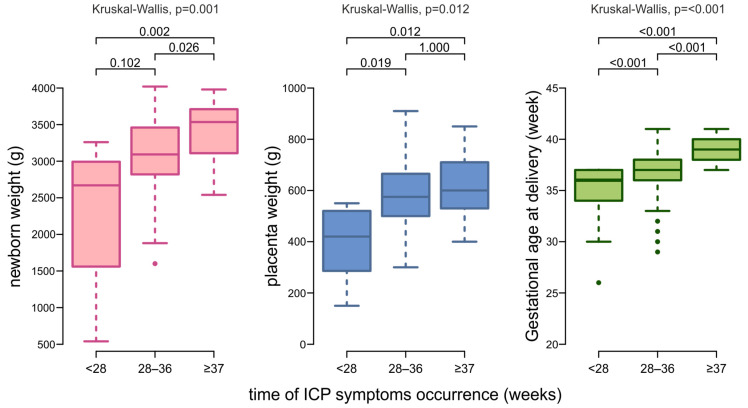
Box plots showing the distribution of data on gestational age at delivery, neonatal weight, and placental weight by the time of ICP symptoms onset.

We observed statistically significant differences in the time of pregnancy termination (*p* < 0.001), newborn weight (*p* = 0.001) and placental weight (*p* = 0.012) between medians in at least two subgroups. Newborns of mothers who developed ICP symptoms before 28 weeks of pregnancy were born earlier—median 36 weeks [IQR: 32.00; 36.50]—with lower birth weights—median 2670 g [IQR: 1560; 2992.5]—and placentas—median 420 g [IQR: 286; 520] (Figure 3). Dunn’s test with Bonferroni correction for multiple comparisons found that the medians of gestational age at delivery differ significantly between all three subgroups (*p* < 0.001). For newborn weights, significant differences were between <28 weeks vs. ≥37 weeks group (*p* = 0.002) and between 28–36 weeks vs. ≥37 weeks (*p* = 0.026). The group with the earliest ICP symptoms differed statistically significantly from the other groups in terms of placental weight: 28–36 weeks (*p* = 0.019) and ≥37 weeks (*p* = 0.012) (Figure 3).

The differences in the results of BA, ALT and AST liver function tests between subgroups distinguished by the time of ICP symptoms occurrence are summarized in Table 4. The serum total bile acid levels increased with the time of onset of ICP symptoms, while serum aminotransferases were opposite. The results of the Kruskal-Wallis rank sum test showed no significant differences between the groups (ALT *p* = 0.145, AST *p* = 0.265, TBA *p* = 0.681).

### 2.5. The Relationship Between the SNVs and Liver Parameters

**Figure 4:**
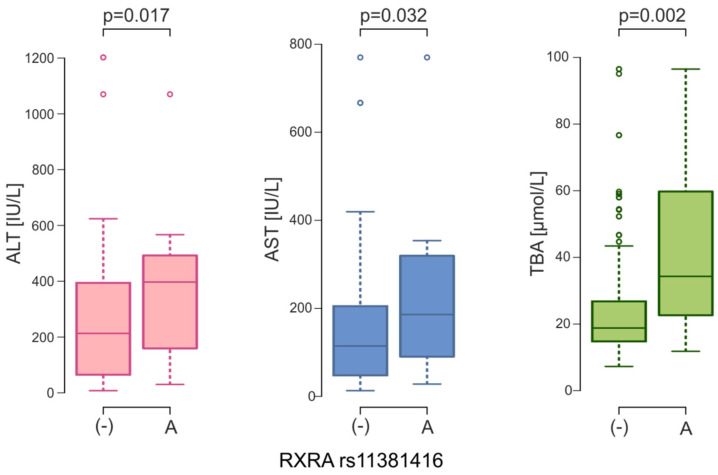
ALT, AST and TBA serum levels between *NR2B1* (*RXRA*) rs11381416 alleles in ICP women (ALT, alanine aminotransferase; AST, aspartate aminotransferase; TBA, total bile acids).

One of the main characteristics of ICP are elevated serum BA and transaminase levels. The Kruskal-Wallis test was conducted to examine the differences in these parameters according to the genotypes in women with ICP. It was observed that genotypes with A insertion of the *NR2B1* rs11381416 variant are associated with an increase in the level of BA (*p* = 0.019), ASP (*p* = 0.079) and ALT (0.060) (Table 5). However, after Dunn’s test with Bonferroni correction was used, no significant differences were found between genotypes. Under the dominant model, significant differences were found for BA (medians for -/- was 18.21 μmol/L [IQR: 14.86; 25.10] vs. 34.30 μmol/L [IQR: 22.35; 58.85] for -/A and -/- genotypes, *p* = 0.008). Comparing the medians for these liver parameters between the (-) and A alleles, the differences were statistically significant. In women with A insertion, an increase in the levels of ALT (397.0 vs. 213.0 IU/L, *p* = 0.017), AST (186.0 vs. 114.4 IU/L, *p* = 0.032), and TBA (34.3 vs. 18.8 μmol/L, *p* = 0.002) was noted (Figure 4).

## 3. Discussion

In pregnancy, physiology changes dramatically; there are increases in plasma lipids, steroid hormones, binding globulins, and numerous metabolic changes to cope with fetal nutritional demands. In healthy pregnant women, total bile acids increase from the first trimester to late pregnancy [33,34,35]. In a study of white Portuguese women, the mean total bile acid concentration was 5.7 ± 0.4 μmol/L in nonpregnant women and slightly higher, although not significantly, in the group of pregnant women (6.6 ± 0.3 μmol/L, ranging from 1.7 μmol/L to 10.4 μmol/L). In contrast, in women with ICP, the mean concentration was 10 times higher and was 62.1 ± 8.2 μmol/L, ranging from 12.3 μmol/L to 219.4 μmol/L [36]. In our study, in healthy women, the median TBAs in serum collected the day after delivery was 1.10 μmol/L [IQR: 0.50; 2.10], and in women with cholestasis measured at diagnosis was 19.75 μmol/L [IQR: 14.93; 33.36]. In a similar study conducted on a population neighboring ours of healthy women from Germany (N = 38), serum bile acid postpartum examinations (conducted in the same way as in our case, 24 h after delivery) were 2.3 ± 1.2 µmol/L (in our case, the average was 1.71 ± 1.68 μmol/L), and in ICP (N = 98) patients in the third trimester, they were 39.0 ± 29.4 µmol/L (in our ICP women at diagnosis, mean ± SD was 29.84 ± 28.06 μmol/L) [37].

The prevalence of ICP has been reported to vary widely across populations and is more common in South Asia, South America, and Scandinavia [38]. Differences between populations are also noted by examining the levels of bile salts in serum. Black women had non-fasting TBA 25.8% higher (95% CI: 9.6–44.4%, *p* = 0.001) than white women, and 24.3% higher (95% CI: 5.7–46.1%, *p* = 0.008) than South Asian women. Levels from South Asian women were similar to those of white women (1.22% higher, 95% CI: 12.1 to 16.5%, *p* = 0.866) [39]. Sometimes even studies in the same population yield different results. For example, the study by Agarwal et al. [40] showed that pregnant Asian-Indian women have higher serum BA levels and therefore suggest that levels > 30 μmol/L can be considered as a cut-off point for diagnosing ICP in this geographical region [40]. In contrast, the study by Yadav et al. [41] reported that serum BA levels in healthy Indian non-pregnant and pregnant women are similar to those in other populations and can be used to diagnose ICP with an optimal cut-off being 8.6 μmol/L [41]. In a study of serum bile acid concentrations in European and South Asian women with or without GDM, Schoonejans et al. [42] found that serum BA homeostasis in late pregnancy was influenced by body mass index and GDM in an ethnicity-specific manner [42]. The incidence of ICP is reported to be 1% of all pregnancies in the European population, with a high occurrence in Sweden (1.5%). All our patients were of Caucasian race and from the Greater Poland Voivodship. Interestingly, in Poland, the estimated rate of ICP is 1.5%, but this is based on few studies on small populations [43,44,45].

Studies show that bile salt concentrations and the incidence of ICP depend on the season. A seasonal pattern for both fasting and postprandial total serum bile acid concentrations was observed in healthy pregnant women from Italy. The highest values measured in the winter season, declining during spring and summer, and with minimum values were measured in the autumn (*p* < 0.01 and 0.02, respectively) [46]. Studies from Scandinavia and Chile have shown evidence of seasonality in ICP, especially higher risks for colder months [47,48]. These results seem contradictory to Sanhal et al.’s [49] findings. In this study from Turkey, the number of new ICP cases was significantly lower in winter and higher in spring. The authors point out that the studies were conducted in lands belonging to different groups in the Köppen climate classification, which may influence the results [49]. The observed ethnic and seasonal differences in the incidence of ICP and in bile salt concentrations may be partly due to variability in diets. In the study by Trefflich et al. [50], fecal and serum bile acid concentrations were examined in 36 vegans and 36 omnivores. Serum primary and glycine-conjugated bile acids were higher in vegans than in omnivores (*p* ≤ 0.01). All fecal bile acids were significantly lower in vegans than in omnivores (*p* < 0.01). These results suggest that animal products and fat, in particular, may increase fecal bile acid levels and increase the risk of colorectal cancer [50]. It is also known that some plant compounds interact with bile acids during digestion in the small intestine. Furthermore, studies indicate the interactions of bile acids with the gut microbiota and their role in bile acid metabolism [51].

The etiology of ICP is complex, with some evidence pointing to genetic factors (familial clustering, increased risk of ICP in siblings of affected women, and increased incidence in certain ethnic groups, e.g., Chilean Araucanians) [52,53]. Most published studies of genetic variants associated with ICP etiopathogenesis have focused on the adenosine triphosphate-binding cassette (ABC) transporters family, mainly the *ABCB4* gene coding for the multidrug resistance 3 (MDR3) protein and the *ABCB11* that encodes bile salt export pump (BSEP) [53,54]. MDR3 translocates phosphatidylcholine across the hepatocanalicular membrane, whereas BSEP transports bile acids from the cytoplasm of the hepatocyte into the canaliculus. Polymorphisms were also studied in *ABCC2* genes (coding multidrug resistance-associated protein 2—MRP2), and *ATP8B1* (which encodes the familial intrahepatic cholestasis 1 protein—FIC1) in women with ICP [55,56,57].

Polymorphic variants of genes from the nuclear receptor family were also analyzed in ICP. Cases of genetic variation in the *NR1I2* gene (encoding pregnane X receptor—PXR) have been reported in South American women [58]. The central role in bile acid homeostasis is played by the nuclear farnesoid receptor (FXR). Van Mill and coworkers [58] have analyzed sequencing data of 92 British ICP cases of mixed ethnicity and identified four heterozygous *FXR* variants. Two of them (rs61755050 and rs56163822) occur in Caucasians and were associated with reduced FXR activity, but only rs61755050 was significantly associated with a higher frequency of ICP (OR = 3.2; 95% CI: 1.1–11.2; *p* = 0.02). In the case of the rs56163822 variant, similarly to our results, no direct association with the etiology of ICP was demonstrated [59]. Upon bile acid activation, FXR binds heterodimerically with another nuclear receptor, retinoid X receptor. RXRA encoded by the *NR2B1* gene participates in different metabolic pathways, but the influence of genetic variants in *RXRA* is poorly studied. In the study by Lima et al. (2013) [60], rs11381416 was genotyped in 622 healthy subjects from a Southern Brazilian community. Carriers of the A allele of the *RXRA* rs11381416 polymorphism have higher TG levels (1.80 ± 1.20 mmol/L) than those without an A insertion (1.52 ± 1.02 mmol/L; *p* = 0.020) [60]. In our ICP women with A insertion, we observed an increase in the levels of ALT (397.0 vs. 213.0 IU/L, *p* = 0.017), AST (186.0 vs. 114.4 IU/L, *p* = 0.032), and TBA (34.3 vs. 18.8 μmol/L, *p* = 0.002). We did not have detailed lipid profiles of women with ICP, which may show other associations between this *RXRA* gene variant and dyslipidemia in cholestatic patients. The relationship between ICP and abnormal lipid profiles was observed already in 1973 [61]. A meta-analysis of 786 participants by Zhan et al. (2022) revealed a significant association between ICP and maternal dyslipidemia, with elevated levels of triglycerides, total cholesterol, low-density lipoprotein cholesterol, and reduced high-density lipoprotein cholesterol levels vs. normal pregnancies [62].

The next nuclear receptor we studied was PPARA, which, like FXR, forms heterodimers with nuclear RXR after ligand binding. PPARA plays a central role in the regulation of multiple metabolic processes. When activated, it induces expression of genes involved in fatty acid uptake, reduction in triglyceride levels, and an increase in high-density lipoprotein expression [63,64,65]. Both nuclear receptors FXR and PPARA provide an intriguing, coordinated response to maintain energy balance in the liver depending on the nutritional status of the body. FXR is activated in the fed state by bile acids returning to the liver, while PPARA is activated in the fasted state in response to the free fatty acids produced by adipocyte lipolysis [24,66]. *PPARA* L162V polymorphism, associated with reduced gene activity, has important effects in dyslipidemia. The V162 minor allele is associated with an increase in triglycerides, total cholesterol, LDL, ApoB, ApoC3, the risk of type 2 diabetes, and a decrease in HDL [67,68]. In our study, no statistically significant association was observed between this *PPARA* gene variant and ICP risk.

Nuclear receptors modulate the activity of the key enzyme for bile acid synthesis, CYP7A1. Our results indicate an association between *CYP7A1* rs3808607 and the risk of ICP, and an association of *NR2B1* rs11381416 with higher liver function test values in women with pregnancy cholestasis. Additionally, individuals with the genotypes with the A duplication of the rs11381416 variant were 3.19 times more frequently observed in women with moderate to severe ICP (with TBA ≥ 40 μmol/L). To better understand the association between these variants and ICP, we searched available databases. In the National Center for Biotechnology Information (NCBI) ClinVar database [69], rs11381416 is not recorded, whereas rs3808607 is classified as “benign” based on two reports (VCV001229509.5, accessed 16 July 2025) [69]. It is also unclear whether and how these variants change the structure of proteins. The rs3808607 variant is located at the 5′ end of the gene, whereas rs11381416 is an intronic single-nucleotide duplication between exons 8 and 9. We could not find any studies on functional consequences (e.g., quantitative effects on gene expression, alternative splicing, functional effects on protein) based on experimental evidence for rs11381416. An interesting study in the case of *CYP7A1* rs3808607 was conducted by Wang et al. [70]. They noted inconsistent research results regarding the rs3808607 variant. On the one hand, the G allele exhibits higher transcriptional activity than the T allele in reporter gene assays. However, association studies link the G allele to increased risk of atherosclerosis or greater lipid levels, which are predicted outcomes of reduced *CYP7A1* activity. Furthermore, rs3808607 does not show a significant association with lipids in GWAS studies. Their study indicates the existence of two interacting variants, rs3808607 and rs9297994, which modulate *CYP7A1* expression and are associated with the risk of coronary heart disease and diabetes. The functional rs9297994, frequently found in Caucasians and located in the downstream region of the *CYP7A1* enhancer, is in strong linkage disequilibrium with the promoter SNP rs3808607 and exerts opposing effects on *CYP7A1* mRNA expression. The minor G allele of rs3808607 is slightly more common than the minor G allele of rs9297994 in the European population (0.43 vs. 0.37), with a drastically different frequency in African Americans (rs3808607 MAF = 0.61, rs9297994 MAF = 0.035). This suggests that African Americans tend to have higher *CYP7A1* activity than Europeans. In our study, the T allele of the rs3808607 variant was a protective factor against ICP risk (OR = 0.697, *p* = 0.038). The G allele, associated with higher *CYP7A1* transcriptional activity and predicted higher BA concentrations, predominated in the ICP group. It is possible that more details regarding the association between *CYP7A1* polymorphic variants and ICP could be obtained by analyzing the rs3808607 variant in combination with rs9297994.

ICP is associated with an increased risk of perinatal complications (premature birth, respiratory disorders, even stillbirth) [71]. We observed statistically significant differences between groups based on the time of onset of ICP symptoms for the mean values of the time of termination of pregnancy (*p* < 0.001), newborn weight (*p* < 0.001), and placental weight (*p* = 0.003). In the meta-analysis by Li et al. [72], the effect of ICP on neonatal weight was assessed, and a lower birth weight was found in neonates with ICP pregnancies compared with normal pregnancies. Moreover, similarly to our study group, early ICP was associated with lower birth weight than late ICP. Pooled data from two studies [73] indicated that the birth weight in the late-onset ICP group was heavier than in the early onset ICP group (WMD: 267 g, 95% CI: 168, 366) [72].

## 4. Materials and Methods

### 4.1. Study Population

A total of 311 subjects were enrolled in this hospital-based case-control study, comprising 96 ICP cases and 211 controls between 2018 and 2020. The study complied with ethical requirements and has been approved by the Ethics Committee of the Poznan University of Medical Sciences (No 842/13). Blood samples were obtained from the Department of Perinatology of Poznan University of Medical Sciences, Poznan.

Ninety-six women in a singleton pregnancy, of Caucasian race and Polish nationality, were qualified to the study group. ICP was recognized on the basis of the following clinical and laboratory criteria: itching (without rash) and/or an increase in TBA value in serum  ≥10 µmol/L on an empty stomach, increase in transaminases ALT and AST (>30 IU/L), and the resolution of symptoms within 2–3 weeks after delivery. Exclusion criteria of the study group contained existence of chronic liver diseases, viral hepatitis, autoimmune diseases, biliary obstruction, gestational hypertension or fetal anomalies. The controls consisted of pregnant women with no maternal diseases or fetal risks who had similar age and clinical characteristics.

### 4.2. Biochemical Analyses

Blood for testing was collected from pregnant women diagnosed with ICP before treatment with ursodeoxycholic acid (UDCA). The concentration of bile acids, total bilirubin, and aspartate and alanine aminotransferases was determined in the blood serum of women with ICP. In a group of healthy pregnant women, bile acid concentrations were determined after delivery. Roche Cobas test BILT3 (Cat. No. 08056960190, Roche Diagnostics, Mannheim, Germany) was used to quantify total bilirubin. Serum ALT and AST were measured with pyridoxal phosphate activation as suggested by the International Federation of Clinical Chemistry and Laboratory Medicine (IFCC II) using Roche tests ALTP2 (Cat. No. 08104697190, Roche Diagnostics, Mannheim, Germany) and ASTP2 (Cat. No. 08104719190, Roche Diagnostics, Mannheim, Germany). The total volume of bile acids in serum was determined using the 5th Generation Enzymatic Colorimetric RX Series kit (Cat. No. BI3863, Randox Laboratories Ltd., Crumlin, UK). The measurements were conducted on a Cobas Pro analyzer (Roche Diagnostics, Basel, Switzerland). All tests were carried out at the Central Laboratory Gynecology and Obstetrics Clinical Hospital, University of Medical Sciences, Poznan, Poland, a certified facility meeting the criteria of ISO 9001:2015-10 (certificate number J-779/13/2023) [74].

### 4.3. DNA Extraction

2 mL ethylenediaminetetraacetic acid anticoagulated peripheral blood samples were collected from all subjects. Genomic DNA was extracted by the QIAamp DNA Blood Mini Kit (Cat. No.: 69506, Qiagen, Hilden, Germany). Nanodrop 2000 Spectrophotometer (Thermo Scientific, Waltham, MA USA) was used to evaluate the concentration (A260) and purity (A260/A280 absorbance ratio) for each DNA sample.

### 4.4. Selection of the SNVs

**Figure 5:**
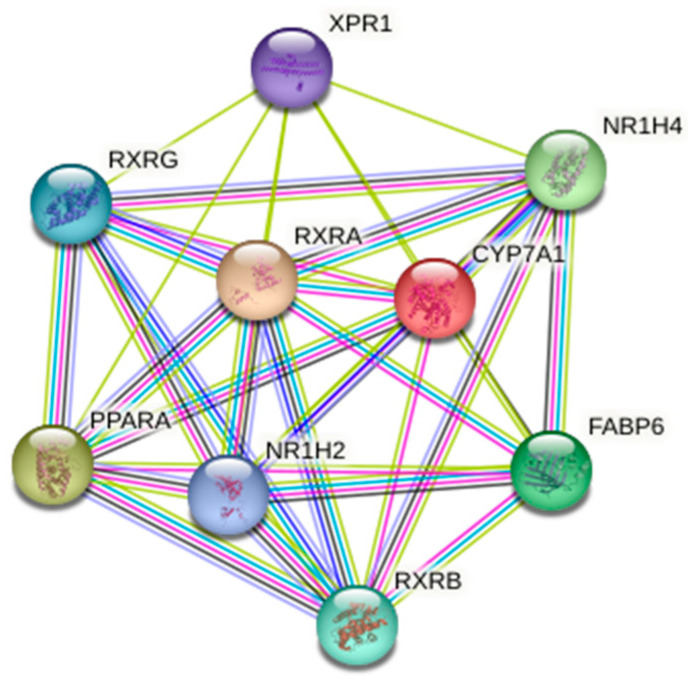
The network of the genes we studied (*CYP7A1*, *NR1H4*, *PPARA*, *RXRA*) and their closest functional partners. The plot was from the STRING (Search Tool for the Retrieval of Interacting Genes) database (http://string-db.org/—accessed on 2 October 2024).

Figure 5 shows the network of connections between the genes we analyzed and their closest neighboring proteins drawn by the STRING database version 12.0 accessed on 2 October 2024 [75].

In this study, five polymorphic variants in four genes regulating bile acid biosynthesis were selected: rs3808607 (*CYP7A1*), rs56163822 (*NR1H4*), rs1800206 (*PPARA*), rs749759, and rs11381416 (*RXRA*). Basic information about the analyzed SNVs and allele frequency distributions in European populations from the 1000Genomes Project is presented in Table 6.

Genotyping was performed by polymerase chain reaction/restriction fragment length polymorphism (PCR/RFLP) using methods described in the previous literature [76,77,78,79,80]. The primers and restriction enzymes used for the RFLP reactions are presented in Table 7. DNA amplification was performed in a 15 μL volume containing 50 ng genomic DNA, 0.25 μM of each primer, 1 U of Taq DNA polymerase and respective buffer (DreamTaq Green DNA polymerase, Cat. No.: EP0712, Thermo Fisher Scientific, Waltham, MA, USA), and 200 μM of each deoxynucleotide (Cat. No. R0241, Thermo Fisher Scientific, Waltham, MA, USA). Samples were denatured in a thermocycler (DNA Engine Dyad^®^ Thermal Cycler, Bio-Rad Laboratories, Inc., Hercules, CA, USA). The variants were detected by restriction analysis using Thermo Fisher Scientific (Waltham, MA, USA) enzymes: BseGI (Cat. No. ER0871), Eco31I (Cat. No. ER0291), BstXI (Cat. No. ER1021), EcoO109I (Cat. No. ER0261), BglI (Cat. No. ER0071). Products were analyzed by electrophoresis on agarose gel with Midori Green Advanced DNA Stain (Cat. No. MG04, Nippon Genetics, Düren, Germany). Positive and negative controls were used in the reactions. 10% of genotype determinations were repeated in independent experiments with complete agreement.

### 4.5. Statistical Analysis

All statistical analyses were conducted in the R programming environment (version 4.3.1, The R Foundation for Statistical Computing, https://cran.r-project.org accessed on 25 November 2024) [81]. For continuous variables, the Shapiro-Wilk test was used to verify normality. Normally distributed variables were expressed as mean ± standard deviation (SD), and in the absence of normal distribution as median and interquartile range (IQR). Nominal variables are presented as observation counts and percentage. The chi-square test or Fisher’s exact test was used for nominal scales, and the *t*-test or Mann-Whitney U test for ordinal scales. Comparisons between a larger number of groups were made using the Kruskal–Wallis test with the post hoc Dunn–Bonferroni comparison procedure. Genotype and allele frequency distributions, in addition to HWE probabilities, were determined applying different models of inheritance (codominant, dominant, recessive, over-dominant, and log-additive) using the SNPassoc package version 2.1-0 [82]; *p* < 0.05 were considered statistically significant.

## 5. Conclusions

In conclusion, normal pregnancy is characterized by metabolic changes that lead to elevated serum bile acid levels and dyslipidemia. Nuclear receptors, due to their ability to translate nutritional signals into gene expression, play an important role in regulating metabolic pathways that influence changes in these parameters. Disruptions in nuclear receptor signaling caused by polymorphic variants in the genes encoding them can result in pregnancy disorders such as ICP. Although the importance of nuclear receptors in bile acid metabolism is crucial, the influence of genetic variants on ICP is poorly studied. Our study suggests that polymorphisms in *CYP7A1* and *RXRA* (*NR2B1*) genes may affect bile acid metabolism and ICP. However, there are several limitations to consider in our study. First, we analyzed only five SNVs of genes related to bile acid metabolism. Moreover, the obtained data were based on a small number of samples, and thus the results of this work must be interpreted cautiously. Future studies should also take into account the influence of environmental factors, especially the diet of the patients. A more complete understanding of the nutrients’ regulatory effects on metabolism via nuclear receptors may provide new insights into the pathophysiology and treatment of ICP.

## Figures and Tables

**Table 1:** Demographic and main clinical data of ICP patients and controls.

Parameter	Controls (N = 211)	ICP (N = 96)	*p*
Maternal age, (years) (mean ± SD)	30.68 ± 4.67	30.43 ± 4.24	0.648
<25, n (%)	19 (9.00%)	8 (8.33%)	0.853
25–34, n (%)	145 (68.72%)	69 (71.88%)	
≥35, n (%)	47 (22.27%)	19 (19.79%)	
Systolic BP, mmHg (median (IQR))	105 [105; 115]	110 [100; 120]	0.166
Diastolic BP, mmHg (median (IQR))	70 [60; 70]	70 [60; 75]	0.206
Maternal pre-pregnancy BMI (kg/m^2^) (median (IQR))	21.14 [19.63; 23.42]	21.95 [20.37; 23.63]	0.109
Underweight (<18.5)	22 (10.43%)	9 (9.38%)	0.775
Normal weight (18.5 ≤ BMI < 25.0)	161 (76.30%)	70 (72.92%)	
Overweight (25.0 ≤ BMI < 30)	21 (9.95%)	13 (13.54%)	
Obesity (≥30)	7 (3.32%)	4 (4.17%)	
Maternal BMI at delivery (kg/m^2^) (median (IQR))	26.72 [24.64; 29.07]	26.23 [23.95; 28.94]	0.131
BMI Increase During Pregnancy (kg/m^2^) (median (IQR))	5.21 [4.15; 6.52]	4.11 [2.94; 5.02]	<0.001
Gestational weight gain, n (%)			
Inadequate	52 (24.64%)	40 (41.67%)	0.010
Adequate	87 (41.23%)	32 (33.33%)	
Excessive	72 (34.12%)	24 (25.00%)	
Number of pregnancies, median (IQR)	2 [1; 2]	2 [1; 2]	0.103
Gestational age at delivery (week), median (IQR)	39 [38; 40]	37 [36; 39]	<0.001
Delivery, n (%)			
Preterm < 37 week	19 (9.00%)	54 (56.25%)	<0.001
Term ≥ 37 week	192 (91.00%)	42 (43.75%)	
Type of delivery, n (%)			0.037
Operative	69 (32.70)	44 (45.83)	
Vaginal	142 (67.30)	52 (54.17)	
Newborn sex, n (%)			0.325
Female	89 (42.18)	47 (48.96)	
Male	122 (57.82)	49 (51.04)	
Newborn weight (g), (mean ± SD)	3425.59 ± 433.82	3091.03 ± 634.81	<0.001
Placenta weight (g), (mean ± SD)	620.18 ± 111.37	581.06 ± 150.63	0.053
TB (mg/dL), median [IQR]	—	0.475 [0.330; 0.720]	—
ALT (IU/L), median [IQR]	—	227.50 [67.45; 399.45]	—
AST (IU/L), median [IQR]	—	124.00 [51.05; 211.95]	—
TBA (μmol/L), median [IQR]	1.10 [0.50; 2.10]	19.75 [14.93; 33.36]	<0.001
ICP classification, n (%)	—		—
low (10 ≤ TBA < 40 μmol/L),		76 (79.17)	
moderate (40 ≤ TBA < 100 μmol/L)		17 (17.71)	
high (TBA ≥ 100 μmol/L)		3 (3.12)	
Onset of ICP symptoms (week), median (IQR)	—	34.00 [30.00; 36.00]	—

Abbreviations: ALT, alanine aminotransferase; AST, aspartate aminotransferase; BMI, body mass index; ICP, Intrahepatic cholestasis of pregnancy; TB, total bilirubin; TBA, total bile acids; gestational weight gain was calculated based on IOM recommendations (2009); ICP classification according to the guidelines of Royal College of Obstetricians and Gynaecologists (RCOG).

**Table 2:** Allelic distribution of SNVs in ICP patients and healthy women.

Gene/SNV	Allels	Controls (N = 422)	ICP (N = 192)	OR (95% CI)	χ^2^	*p*
*NR1H4* rs56163822	G	414 (0.981)	187 (0.973)	1.383 (0.446–4.286)	0.319	0.571
	T	8 (0.018)	5 (0.026)			
*CYP7A1* rs3808607	G	184 (0.436)	101 (0.526)	0.697 (0.495–0.981)	4.299	0.038
	T	238 (0.563)	91 (0.473)			
*NR2B1* rs749759	G	316 (0.748)	139 (0.723)	1.136 (0.773~1.671)	0.424	0.514
	A	106 (0.251)	53 (0.276)			
*NR2B1* rs11381416	(-)	392 (0.928)	175 (0.911)	1.269 (0.682~2.362)	0.568	0.450
	A	30 (0.071)	17 (0.088)			
*PPARA* rs1800206	C	397 (0.94)	174 (0.906)	1.642 (0.873~3.089)	2.412	0.120
	G	25 (0.059)	18 (0.093)			

**Table 3:** Association between single nucleotide variants and ICP risk under different genetic models.

Inheritance Model	Genotypes	Controls (N = 211)	ICP (N = 96)	OR (95% CI)	*p*	AIC
*NR1H4* rs56163822						
Codominant	GG	203 (96.2)	91 (94.8)	1.00	0.574	385.1
	GT	8 (3.8)	5 (5.2)	1.39 (0.44–4.38)		
	TT	0 (0.0)	0 (0.0)	—		
log-Additive	0, 1, 2	211 (68.7)	96 (31.3)	1.39 (0.44–4.38)	0.574	385.1
*CYP7A1* rs3808607						
Codominant	GG	42 (19.9)	30 (31.2)	1.00	0.093	382.7
	GT	100 (47.4)	41 (42.7)	0.57 (0.32–1.04)		
	TT	69 (32.7)	25 (26.0)	0.51 (0.26–0.98)		
Dominant	GT + TT	169 (80.1)	66 (68.8)	0.55 (0.32–0.95)	0.032	380.9
Recessive	GG + GT	142 (67.3)	71 (74.0)	0.72 (0.42–1.24)	0.236	384.0
Overdominant	TT + GG	111 (52.6)	55 (57.3)	1.21 (0.74–1.97)	0.445	384.9
log-Additive	0, 1, 2	211 (68.7)	96 (31.3)	0.71 (0.51–1.00)	0.046	381.4
*NR2B1* rs749759						
Codominant	GG	104 (53.1)	48 (54.5)	1.00	0.779	357.1
	AG	78 (39.8)	32 (36.4)	0.89 (0.52–1.52)		
	AA	14 (7.1)	8 (9.1)	1.24 (0.49–3.15)		
Dominant	AG-AA	92 (46.9)	40 (45.5)	0.94 (0.57–1.56)	0.817	355.5
Recessive	GG + AG	182 (92.9)	80 (90.9)	1.30 (0.52–3.22)	0.575	355.3
Overdominant	GG + AA	118 (60.2)	56 (63.6)	0.86 (0.51–1.45)	0.582	355.3
log-Additive	0, 1, 2	196 (69.0)	88 (31.0)	1.01 (0.68–1.50)	0.955	355.6
*NR2B1* rs11381416						
Codominant	-/-	182 (86.3)	81 (84.4)	1.00	0.447	385.8
	-/A	28 (13.3)	13 (13.5)	1.04 (0.51–2.12)		
	A/A	1 (0.5)	2 (2.1)	4.49 (0.40–50.27)		
Dominant	-/A+ -/-	29 (13.7)	15 (15.6)	1.16 (0.59–2.29)	0.665	385.3
Recessive	A/A + -/A	210 (99.5)	94 (97.9)	4.47 (0.40–49.88)	0.206	383.8
Overdominant	A/A + -/-	183(86.7)	83(86.5)	1.02 (0.50 2.08)	0.948	385.4
log-Additive	0, 1, 2	211 (68.7)	96 (31.3)	1.25 (0.68–2.29)	0.468	384.9
*PPARA* rs1800206						
Codominant	CC	187 (88.6)	79 (82.3)	1.00	0.323	385.2
	CG	23 (10.9)	16 (16.7)	1.65 (0.83–3.28)		
	GG	1 (0.5)	1 (1.0)	2.37 (0.15–38.32)		
Dominant	CG + GG	24 (11.4)	17 (17.7)	1.68 (0.85–3.29)	0.138	383.2
Recessive	CC + CG	210 (99.5)	95 (99.0)	2.21 (0.14–35.72)	0.581	385.1
Overdominant	CC + GG	188 (89.1)	80 (83.3)	1.63 (0.82–3.26)	0.168	383.5
log-Additive	0, 1, 2	211 (68.7)	96 (31.3)	1.63 (0.87–3.05)	0.134	383.2

AIC, Akaike information criteria; ICP, Intrahepatic cholestasis of pregnancy; OR, odds ratio; 95% CI, 95% confidence interval. *p* < 0.05 was considered significant.

**Table 4:** Differences in liver function tests BA, ALT, and AST between subgroups distinguished by the time of ICP symptoms occurrence.

Parameter	<28 Weeks (N = 9)	28–36 Weeks (N = 69)	≥37 Weeks (N = 18)	*p*
ALT (IU/L), median [IQR]	371.10 [249.75; 396.65]	213.00 [78.10; 409.00]	62.80 [17.70; 320.45]	0.145
AST (IU/L), median [IQR]	208.80 [114.60; 225.55]	114.40 [57.90; 196.00]	36.50 [25.55; 179.00]	0.265
TBA (μmol/L), median [IQR]	19.80 [15.00; 22.90]	19.70 [14.86; 34.80]	18.90 [15.00; 28.04]	0.681

ALT, alanine aminotransferase; AST, aspartate aminotransferase; TBA, total bile acids; p, Kruskal-Wallis rank sum test.

**Table 5:** Association between genotypes with median serum TBA, AST and ALT values in ICP women.

Gene/SNV	Genotypes	TBA (μmol/L)	*p*	ALT (IU/L)	*p*	AST (IU/L)	*p*
*NR1H4* rs56163822	GG (N = 91)	21.20 [15.00; 33.86]	0.091	237.10 [68.00; 400.20]	0.132	131.00 [52.00; 215.10]	0.117
	GA (N = 5)	13.72 [12.10; 18.21]		59.85 [12.70; 107.00]		41.20 [14.40; 68.00]	
	AA (N = 0)	—		—		—	
*CYP7A1* rs3808607	GG (N = 30)	22.05 [12.81; 34.30]	0.619	271.00 [148.40; 414.90]	0.130	134.40 [85.30; 236.00]	0.209
	GT (N = 41)	17.54 [14.86; 25.41]		156.50 [32.60; 318.00]		103.20 [28.95; 178.50]	
	TT (N = 25)	21.20 [16.20; 28.40]		245.60 [78.10; 398.70]		123.45 [48.30; 303.40]	
*NR2B1* rs749759	GG (N = 53)	22.30 [15.40; 33.30]	0.623	242.10 [68.00; 398.70]	0.911	132.00 [50.10; 215.10]	0.982
	GA (N = 33)	17.60 [15.00; 24.97]		179.50 [63.50; 352.75]		108.00 [55.00; 187.05]	
	AA (N = 10)	17.71 [11.49; 54.28]		265.95 [30.00; 427.30]		122.35 [28.00; 307.50]	
*NR2B1* rs11381416	-/- (N = 81)	18.21 [14.86; 25.10]	0.019	213.00 [61.65; 382.10]	0.060	113.20 [48.10; 208.80]	0.079
	-/A ((N = 13)	25.41 [22.00; 58.00]		277.60 [135.65; 403.00]		171.30 [72.00; 193.00]	
	A/A (N = 2)	65.40 [34.30; 96.50]		529.20 [491.60; 566.80]		336.40 [319.00; 353.80]	
*PPARA* rs1800206	CC (N = 79)	18.37 [14.30; 30.85]	0.524	239.20 [72.50; 399.45]	0.239	124.00 [53.10; 211.95]	0.232
	CG (N = 16)	22.52 [16.35; 36.44]		118.55 [66.85; 249.20]		108.00 [51.05; 149.25]	
	GG (N = 1)	24.80 [24.80; 24.80]		506.10 [506.10; 506.10]		419.40 [419.40; 419.40]	

ALT, alanine aminotransferase; AST, aspartate aminotransferase; TBA, total bile acids; Median (IQR).

**Table 6:** Characteristics of selected genes and variants.

Gene	rs Number	Position (GRCh38.p14)	Region	Allele	Allele Frequency in Europe *
*NR1H4*	rs56163822	chr12:100493323	Non Coding Transcript Variant	G > T	G = 0.98, T = 0.02
*CYP7A1*	rs3808607	chr8:58500365	2KB Upstream Variant	G > T	G = 0.43, T = 0.57
*NR2B1*	rs749759	chr9:134432806	Intron Variant	A > G	A = 0.23, G = 0.77
*NR2B1*	rs11381416	chr9:134432216	Intron Variant	(-) > A	(-) = 0.92, A = 0.08
*PPARA*	rs1800206	chr22:46218377	Missense Variant (p.Leu162Val)	C > G	C = 0.94, G = 0.06

SNV ID in database dbSNP; given name according to NCBI; * 1000Genomes Project.

**Table 7:** PCR primers and restriction enzymes used for SNV genotyping.

Gene	SNV	Primer Sequences	Restriction Enzyme
*NR1H4*	rs56163822	5′-GCATTCCCACAGTCACAAAC-3′ 5′-TGAGGAAATGCCTAGATGATGA-3′	BseGI
*CYP7A1*	rs3808607	5′-AATTAGCCATTTGTTCATTCTATTAG-3′ 5′-AATGTTTTTCCCAGTTCTCTTTC-3′	Eco31I
*NR2B1*	rs749759	5′-ATAGGGCTTGCCTGCCTAGA-3′ 5′-CTCCACCATAGCCCAAGTGA-3′	BstXI
*NR2B1*	rs11381416	5′-GCCTCCTCCTGGCTGTACTT-3′ 5′-CTCATGACAACTGCCTTGCT-3′	EcoO109I
*PPARA*	rs1800206	5′-AACAATAAGTGAGCAACAAAAAAG-3′ 5′-CGTTGTGTGACATCCCGCCAGAAA-3′	BglI

## Data Availability

The data presented in this study are available on request from the corresponding author.

## References

[1] Malarkiewicz P., Nowacka U., Januszaniec A., Mankiewicz A., Kozłowski S., Issat T. (2024). Intrahepatic Cholestasis of Pregnancy during COVID-19 Pandemic. Medicina.

[2] Lee R.H., Greenberg M., Metz T.D., Pettker C.M. (2021). Society for Maternal-Fetal Medicine Consult Series #53: Intrahepatic cholestasis of pregnancy: Replaces Consult #13, April 2011. Am. J. Obstet. Gynecol..

[3] Floreani A., Caroli D., Lazzari R., Memmo A., Vidali E., Colavito D., D’Arrigo A., Leon A., Romero R., Gervasi M.T. (2013). Intrahepatic cholestasis of pregnancy: New insights into its pathogenesis. J. Matern.-Fetal Neonatal Med..

[4] Glantz A., Marschall H.U., Mattsson L.A. (2004). Intrahepatic cholestasis of pregnancy: Relationships between bile acid levels and fetal complication rates. Hepatology.

[5] Kawakita T., Parikh L.I., Ramsey P.S., Huang C.C., Zeymo A., Fernandez M., Smith S., Iqbal S.N. (2015). Predictors of adverse neonatal outcomes in intrahepatic cholestasis of pregnancy. Am. J. Obstet. Gynecol..

[6] Majsterek M., Wierzchowska-Opoka M., Makosz I., Kreczyńska L., Kimber-Trojnar Ż., Leszczyńska-Gorzelak B. (2022). Bile Acids in Intrahepatic Cholestasis of Pregnancy. Diagnostics.

[7] Pillarisetty L.S., Sharma A. (2024). Pregnancy Intrahepatic Cholestasis. StatPearls [Internet].

[8] Waheed A.M.I., Jaiswal A., Yelne S., Nandanwar V. (2024). Navigating Perinatal Challenges: A Comprehensive Review of Cholestasis of Pregnancy and Its Impact on Maternal and Fetal Health. Cureus.

[9] Zhuo H., Fan J., Yao L., Zheng L., Chai Y. (2023). MDR3 rs2109505 and rs1202283 polymorphisms are associated with susceptibility to intrahepatic cholestasis of pregnancy: A meta-analysis. Adv. Clin. Exp. Med..

[10] Obiegbusi C.N., Dong X.J., Obiegbusi S.C., Jin X., Okoene I.K. (2024). Predictors of Adverse Fetal Outcomes in Intrahepatic Cholestasis of Pregnancy (ICP): A Narrative Review. Reprod. Sci..

[11] Copple B.L., Li T. (2016). Pharmacology of bile acid receptors: Evolution of bile acids from simple detergents to complex signaling molecules. Pharmacol. Res..

[12] Perino A., Schoonjans K. (2022). Metabolic Messengers: Bile acids. Nat. Metab..

[13] Cohen J.C., Cali J.J., Jelinek D.F., Mehrabian M., Sparkes R.S., Lusis A.J., Russell D.W., Hobbs H.H. (1992). Cloning of the human cholesterol 7 alpha-hydroxylase gene (CYP7) and localization to chromosome 8q11–q12. Genomics.

[14] De Castro-Orós I., Pampín S., Cofán M., Mozas P., Pintó X., Salas-Salvadó J., Rodríguez-Rey J.C., Ros E., Civeira F., Pocoví M. (2011). Promoter variant −204A > C of the cholesterol 7α-hydroxylase gene: Association with response to plant sterols in humans and increased transcriptional activity in transfected HepG2 cells. Clin. Nutr..

[15] Sezer E., Demirdöğen C.B., Demirkaya Ş., Bulut G., Akkulak M., Evin E., Adalı O. (2022). Association of cholesterol 7α-hydroxylase (CYP7A1) promoter polymorphism (rs3808607) and cholesterol 24S-hydroxylase (CYP46A1) intron 2 polymorphism (rs754203) with serum lipids, vitamin D levels, and multiple sclerosis risk in the Turkish population. Neurol. Sci..

[16] Lu Y., Feskens E.J., Boer J.M., Müller M. (2010). The potential influence of genetic variants in genes along bile acid and bile metabolic pathway on blood cholesterol levels in the population. Atherosclerosis.

[17] Couture P., Otvos J.D., Cupples L.A., Wilson P.W., Schaefer E.J., Ordovas J.M. (1999). Association of the A-204C polymorphism in the cholesterol 7alpha-hydroxylase gene with variations in plasma low density lipoprotein cholesterol levels in the Framingham Offspring Study. J. Lipid. Res..

[18] Makishima M., Okamoto A.Y., Repa J.J., Tu H., Learned R.M., Luk A., Hull M.V., Lustig K.D., Mangelsdorf D.J., Shan B. (1999). Identification of a nuclear receptor for bile acids. Science.

[19] Li T., Chiang J.Y. (2013). Nuclear receptors in bile acid metabolism. Drug Metab. Rev..

[20] Stojancevic M., Stankov K., Mikov M. (2012). The impact of farnesoid X receptor activation on intestinal permeability in inflammatory bowel disease. Can. J. Gastroenterol..

[21] Marzolini C., Tirona R.G., Gervasini G., Poonkuzhali B., Assem M., Lee W., Leake B.F., Schuetz J.D., Schuetz E.G., Kim R.B. (2007). A common polymorphism in the bile acid receptor farnesoid X receptor is associated with decreased hepatic target gene expression. Mol. Endocrinol..

[22] Acevedo J.M., Hoermann B., Schlimbach T., Teleman A.A. (2018). Changes in global translation elongation or initiation rates shape the proteome via the Kozak sequence. Sci. Rep..

[23] Wilson A., Wang Q., Almousa A.A., Jansen L.E., Choi Y.H., Schwarz U.I., Kim R.B. (2020). Genetic variation in the farnesoid X-receptor predicts Crohn’s disease severity in female patients. Sci. Rep..

[24] Zhou S., You H., Qiu S., Yu D., Bai Y., He J., Cao H., Che Q., Guo J., Su Z. (2022). A new perspective on NAFLD: Focusing on the crosstalk between peroxisome proliferator-activated receptor alpha (PPARα) and farnesoid X receptor (FXR). Biomed. Pharmacother..

[25] Wang N., Zou Q., Xu J., Zhang J., Liu J. (2018). Ligand binding and heterodimerization with retinoid X receptor α (RXRα) induce farnesoid X receptor (FXR) conformational changes affecting coactivator binding. J. Biol. Chem..

[26] Wang Y.D., Chen W.D., Moore D.D., Huang W. (2008). FXR: A metabolic regulator and cell protector. Cell Res..

[27] Christofides A., Konstantinidou E., Jani C., Boussiotis V.A. (2021). The role of peroxisome proliferator-activated receptors (PPAR) in immune responses. Metabolism.

[28] Sun Y., Zhang L., Jiang Z. (2024). The role of peroxisome proliferator-activated receptors in the regulation of bile acid metabolism. Basic Clin. Pharmacol. Toxicol..

[29] Yong E.L., Li J., Liu M.H. (2008). Single gene contributions: Genetic variants of peroxisome proliferator-activated receptor (isoforms alpha, beta/delta and gamma) and mechanisms of dyslipidemias. Curr. Opin. Lipidol..

[30] Mazzotti D.R., Singulane C.C., Ota V.K., Rodrigues T.P., Furuya T.K., de Souza F.J., Cordeiro B.G., Magalhães C., Chen E.S., Jacomini A. (2011). PPARα polymorphisms as risk factors for dyslipidemia in a Brazilian population. Mol. Genet. Metab..

[31] Rasmussen K.M., Yaktine A.L., Institute of Medicine (US) and National Research Council (US), Committee to Reexamine IOM Pregnancy Weight Guidelines (2009). Weight Gain During Pregnancy: Reexamining the Guidelines.

[32] Girling J., Knight C.L., Chappell L. (2022). Royal College of Obstetricians and Gynaecologists. Intrahepatic cholestasis of pregnancy: Green-top Guideline No. 43 June 2022. BJOG.

[33] Soma-Pillay P., Nelson-Piercy C., Tolppanen H., Mebazaa A. (2016). Physiological changes in pregnancy. Cardiovasc. J. Afr..

[34] Gagnon M., Trottier J., Weisnagel S.J., Gagnon C., Carreau A.M., Barbier O., Morisset A.S. (2021). Bile acids during pregnancy: Trimester variations and associations with glucose homeostasis. Health Sci. Rep..

[35] Zhu B., Yin P., Ma Z., Ma Y., Zhang H., Kong H., Zhu Y. (2019). Characteristics of bile acids metabolism profile in the second and third trimesters of normal pregnancy. Metabolism.

[36] Brites D., Rodrigues C.M., van-Zeller H., Brito A., Silva R. (1998). Relevance of serum bile acid profile in the diagnosis of intrahepatic cholestasis of pregnancy in an high incidence area: Portugal. Eur. J. Obstet. Gynecol. Reprod. Biol..

[37] Nees J., Ammon F.J., Mueller J., Fluhr H., Mueller S. (2023). Liver stiffness in pregnant women with intrahepatic cholestasis of pregnancy: A case control study. World J. Hepatol..

[38] Gao X.X., Ye M.Y., Liu Y., Li J.Y., Li L., Chen W., Lu X., Nie G., Chen Y.H. (2020). Prevalence and risk factors of intrahepatic cholestasis of pregnancy in a Chinese population. Sci. Rep..

[39] Mitchell A.L., Ovadia C., Syngelaki A., Souretis K., Martineau M., Girling J., Vasavan T., Fan H.M., Seed P.T., Chambers J. (2021). Re-evaluating diagnostic thresholds for intrahepatic cholestasis of pregnancy: Case-control and cohort study. BJOG.

[40] Agarwal N., Mahey R., Kulshrestha V., Kriplani A., Saraya A., Sachdev V. (2022). Serum Bile Acids in Intrahepatic Cholestasis of Pregnancy (ICP), Versus Pregnant and Nonpregnant Controls in Asian Indian Women and a Proposed Scoring to Optimize Management in ICP. J. Obstet. Gynaecol. India.

[41] Yadav S., Goel A., Lingaiah R., Pradhan M., Katiyar H., Aggarwal R. (2022). Serum Bile Acid Levels in Women With Intrahepatic Cholestasis of Pregnancy in India. J. Clin. Exp. Hepatol..

[42] Schoonejans J.M., Fan H.M., Mitchell A.L., Lövgren-Sandblom A., Sukumar N., Periyathambi N., Weldeselassie Y., Seed P.T., Molinaro A., Marschall H.U. (2024). Serum bile acid measurements in women of European and South Asian ethnicity with or without gestational diabetes mellitus: A cohort study. BJOG.

[43] Geenes V., Williamson C. (2009). Intrahepatic cholestasis of pregnancy. World J. Gastroenterol..

[44] Wójcicka-Jagodzińska J., Kuczyńska-Sicińska J., Czajkowski K., Smolarczyk R. (1989). Carbohydrate metabolism in the course of intrahepatic cholestasis in pregnancy. Am. J. Obstet. Gynecol..

[45] Majewska A., Godek B., Bomba-Opon D., Wielgos M. (2019). Association between intrahepatic cholestasis in pregnancy and gestational diabetes mellitus. A retrospective analysis. Ginekol. Pol..

[46] Huri M., Seravalli V., Lippi C., Tofani L., Galli A., Petraglia F., Di Tommaso M. (2022). Intrahepatic cholestasis of pregnancy—Time to redefine the reference range of total serum bile acids: A cross-sectional study. BJOG.

[47] Berg B., Helm G., Petersohn L., Tryding N. (1986). Cholestasis of pregnancy: Clinical and laboratory studies. Acta Obstet. Gynecol. Scand..

[48] Reyes H., Gonzalez M.C., Ribalta J., Aburto H., Matus C., Schramm G., Katz R., Medina E. (1978). Prevalence of intrahepatic cholestasis of pregnancy in Chile. Ann. Intern. Med..

[49] Sanhal C.Y., Dağlar K., Kara Ö., Kırbaş A., Uygur D., Yücel A. (2016). Seasonal Impact in the Frequency of Intrahepatic Cholestasis of Pregnancy. Gynecol. Obstet Reprod. Med..

[50] Trefflich I., Marschall H.U., Giuseppe R.D., Ståhlman M., Michalsen A., Lampen A., Abraham K., Weikert C. (2019). Associations between Dietary Patterns and Bile Acids-Results from a Cross-Sectional Study in Vegans and Omnivores. Nutrients.

[51] Naumann S., Haller D., Eisner P., Schweiggert-Weisz U. (2020). Mechanisms of Interactions between Bile Acids and Plant Compounds-A Review. Int. J. Mol. Sci..

[52] Dixon P.H., Williamson C. (2008). The molecular genetics of intrahepatic cholestasis of pregnancy. Obstet. Med..

[53] Abu-Hayyeh S., Papacleovoulou G., Williamson C. (2013). Nuclear receptors, bile acids and cholesterol homeostasis series—Bile acids and pregnancy. Mol. Cell. Endocrinol..

[54] Zöllner J., Williamson C., Dixon P.H. (2024). Genetic issues in ICP. Obstet. Med..

[55] Sookoian S., Castaño G., Burgueño A., Gianotti T.F., Pirola C.J. (2008). Association of the multidrug-resistance-associated protein gene (ABCC2) variants with intrahepatic cholestasis of pregnancy. J. Hepatol..

[56] Müllenbach R., Bennett A., Tetlow N., Patel N., Hamilton G., Cheng F., Chambers J., Howard R., Taylor-Robinson S.D., Williamson C. (2005). ATP8B1 mutations in British cases with intrahepatic cholestasis of pregnancy. Gut.

[57] Painter J.N., Savander M., Ropponen A., Nupponen N., Riikonen S., Ylikorkala O., Lehesjoki A.E., Aittomäki K. (2005). Sequence variation in the ATP8B1 gene and intrahepatic cholestasis of pregnancy. Eur. J. Hum. Genet..

[58] Castaño G., Burgueño A., Fernández Gianotti T., Pirola C.J., Sookoian S. (2010). The influence of common gene variants of the xenobiotic receptor (PXR) in genetic susceptibility to intrahepatic cholestasis of pregnancy. Aliment. Pharmacol. Ther..

[59] Van Mil S.W., Milona A., Dixon P.H., Mullenbach R., Geenes V.L., Chambers J., Shevchuk V., Moore G.E., Lammert F., Glantz A.G. (2007). Functional variants of the central bile acid sensor FXR identified in intrahepatic cholestasis of pregnancy. Gastroenterology.

[60] Lima L.O., Almeida S., Hutz M.H., Fiegenbaum M. (2013). PPARA, RXRA, NR1I2 and NR1I3 gene polymorphisms and lipid and lipoprotein levels in a Southern Brazilian population. Mol. Biol. Rep..

[61] Johnson P. (1973). Studies in cholestasis of pregnancy with special reference to lipids and lipoproteins. Acta Obstet. Gynecol. Scand. Suppl..

[62] Zhan Y., Xu T., Chen T., Wang X. (2022). Intrahepatic cholestasis of pregnancy and maternal dyslipidemia: A systematic review and meta-analysis. Acta Obstet. Gynecol. Scand..

[63] Jaffe S., Normand N., Jayaram A., Orfanelli T., Doulaveris G., Passos M., Kanninen T.T., Bongiovanni A.M., Linhares I.M., Witkin S.S. (2013). Unique variation in genetic selection among Black North American women and its potential influence on pregnancy outcome. Med. Hypotheses.

[64] Fruchart J.C. (2009). Peroxisome proliferator-activated receptor-alpha (PPARalpha): At the crossroads of obesity, diabetes and cardiovascular disease. Atherosclerosis.

[65] Changizi Z., Kajbaf F., Moslehi A. (2023). An Overview of the Role of Peroxisome Proliferator-activated Receptors in Liver Diseases. J. Clin. Transl. Hepatol..

[66] Kim K.H., Moore D.D. (2017). Regulation of Liver Energy Balance by the Nuclear Receptors Farnesoid X Receptor and Peroxisome Proliferator Activated Receptor α. Dig. Dis..

[67] Contreras A.V., Torres N., Tovar A.R. (2013). PPAR-α as a key nutritional and environmental sensor for metabolic adaptation. Adv. Nutr..

[68] Ruscica M., Busnelli M., Runfola E., Corsini A., Sirtori C.R. (2019). Impact of PPAR-Alpha Polymorphisms-The Case of Metabolic Disorders and Atherosclerosis. Int. J. Mol. Sci..

[69] National Center for Biotechnology Information ClinVar; [VCV001229509.5]. https://www.ncbi.nlm.nih.gov/clinvar/variation/VCV001229509.5.

[70] Wang D., Hartmann K., Seweryn M., Sadee W. (2018). Interactions Between Regulatory Variants in CYP7A1 (Cholesterol 7α-Hydroxylase) Promoter and Enhancer Regions Regulate CYP7A1 Expression. Circ. Genom. Precis. Med..

[71] Piechota J., Jelski W. (2020). Intrahepatic Cholestasis in Pregnancy: Review of the Literature. J. Clin. Med..

[72] Li L., Chen Y.H., Yang Y.Y., Cong L. (2018). Effect of Intrahepatic Cholestasis of Pregnancy on Neonatal Birth Weight: A Meta-Analysis. J. Clin. Res. Pediatr. Endocrinol..

[73] Zhou L., Qi H.B., Luo X. (2013). Analysis of clinical characteristics and perinatal outcome of early-onset intrahepatic cholestasis of pregnancy. Zhonghua Fu Chan Ke Za Zhi.

[74] (2015). Quality Management Systems—Requirements, 5th ed..

[75] Szklarczyk D., Kirsch R., Koutrouli M., Nastou K., Mehryary F., Hachilif R., Gable A.L., Fang T., Doncheva N.T., Pyysalo S. (2023). The STRING database in 2023: Protein-protein association networks and functional enrichment analyses for any sequenced genome of interest. Nucleic Acids Res..

[76] Hagiwara T., Kono S., Yin G., Toyomura K., Nagano J., Mizoue T., Mibu R., Tanaka M., Kakeji Y., Maehara Y. (2005). Genetic polymorphism in cytochrome P450 7A1 and risk of colorectal cancer: The Fukuoka Colorectal Cancer Study. Cancer Res..

[77] Iwata R., Baur K., Stieger B., Mertens J.C., Daly A.K., Frei P., Braun J., Vergopoulos A., Stickel F., Sabrane K. (2011). A common polymorphism in the ABCB11 gene is associated with advanced fibrosis in hepatitis C but not in non-alcoholic fatty liver disease. Clin. Sci..

[78] Grzegorzewska A.E., Świderska M.K., Mostowska A., Warchoł W., Jagodziński P.P. (2016). Polymorphisms of Vitamin D Signaling Pathway Genes and Calcium-Sensing Receptor Gene in respect to Survival of Hemodialysis Patients: A Prospective Observational Study. Int. J. Endocrinol..

[79] Vasků V., Bienertová Vasků J., Pávková Goldbergová M., Vasků A. (2007). Three retinoid X receptor gene polymorphisms in plaque psoriasis and psoriasis guttata. Dermatology.

[80] Lacquemant C., Lepretre F., Pineda Torra I., Manraj M., Charpentier G., Ruiz J., Staels B., Froguel P. (2000). Mutation screening of the PPARalpha gene in type 2 diabetes associated with coronary heart disease. Diabetes Metab..

[81] R Core Team (2023). R: A Language and Environment for Statistical Computing.

[82] Moreno V., Gonzalez J., Pelegri D. (2022). SNPassoc: SNPs-Based Whole Genome Association Studies. R Package Version 2.1-0. https://CRAN.R-project.org/package=SNPassoc.

